# Guidewire Method for Measuring Local Left Ventricular Electrical Activation Time During Cardiac Resynchronization Implantation

**DOI:** 10.19102/icrm.2018.090102

**Published:** 2018-01-15

**Authors:** Seth J. Rials, Michele Pershing, Christy Collins

**Affiliations:** ^1^OhioHealth Heart and Vascular Physicians, Division of Cardiology, Grant Medical Center, Columbus, OH, USA; ^2^OhioHealth Research Institute, Columbus, OH, USA

**Keywords:** Cardiac resynchronization, guidewire, local ventricular activation time, QLV

## Abstract

The timing of local activation at left ventricular (LV) pacing leads is measured from the onset of the QRS complex to the peak of the LV electrogram (QLV). Pacing from the sites of late activation is associated with higher response rates to cardiac resynchronization therapy (CRT). Prior studies have measured QLV from permanent pacing leads, or have used electroanatomic mapping systems. The current study compares QLV measurements made with a guidewire to those collected from permanent LV pacing leads positioned at the same venous site without the use of electroanatomic mapping systems. In this study, 20 patients undergoing CRT implantation (14 males, mean QRS: 164.0 ms) had QLV measurements taken using a guidewire. QLV and LV electrogram duration measurements were made at LV pacing sites, and were repeated after positioning the permanent LV pacing lead at the same site. There was no difference in QLV measurements obtained using a guidewire and those obtained using the permanent pacing lead placed at the same site (p = 0.569). QLV measurements obtained with a guidewire and the permanent LV pacing lead at the same site, respectively, were strongly correlated (r = 0.965; p < 0.001). The median absolute difference in electrogram duration was 7.0 ms (p = 0.55). The average time required to make QLV measurements using the guidewire was 11.7 minutes [standard deviation (SD): 6.8]. The average total fluoroscopy time for the entire CRT implant procedure was 10.9 minutes (SD: 5.1). In light of these results, it can be suggested that a guidewire can be used to prospectively measure LV prior to selection or placement of a permanent pacing lead without the use of an electroanatomic mapping system.

## Introduction

Cardiac resynchronization therapy (CRT) is a clinically proven method for reducing heart failure admissions and mortality in patients with systolic heart failure and left bundle branch block (LBBB).^1^ Traditional implant techniques target the posterolateral wall of the left ventricle (LV) using a combination of radiographic appearance, pacing parameters, lead stability, and an absence of diaphragmatic stimulation to determine the final lead position. Unfortunately, CRT is associated with a nonresponder rate of 20% to 40%, depending on the definition used for response.^2^

One predictor of response to CRT is the timing of activation measured at the LV electrode relative to the onset of the QRS complex (QLV). Prolonged QLV (QLV > 95 ms) is associated with both an improved acute response to and marked long-term improvement with CRT.^3,4^ Prospective measurement of QLV has been described using three-dimensional (3D) mapping systems in conjunction with a guidewire,^5–7^ and with LV pacing leads^8^; however, this method is limited in that it can only be used by those electrophysiology (EP) laboratories with access to such systems and those who can afford the additional cost and time associated with 3D mapping. To determine if QLV measurements can be made before lead selection and without a 3D mapping system, electrogram recordings were made using a guidewire and a standard EP laboratory recording system, and then compared with recordings obtained from permanent LV pacing electrodes implanted at the same site.

## Methods

Clinical data, electrograms, and venograms were retrospectively analyzed from 20 patients who had indications either for initial CRT device implantation, or for an upgrade to a CRT [either a CRT-defibrillator (CRT-D) or a CRT-pacemaker (CRT-P)] device. Following insertion of a right ventricular (RV) lead (in new implant patients), coronary sinus cannulation and retrograde occlusive venography were performed. Telescoping sheaths and a guidewire (either Kinetix™; Boston Scientific, Natick, MA, USA, or BMW; Abbott Vascular, Santa Clara, CA, USA) were inserted in a standard fashion. The sheaths and guidewire were used to cannulate target veins of interest and to measure QLV intervals. QLV measurements were made in a unipolar configuration from the tip of the guidewire (cathode), to a retractor in the surgical pocket (anode/ground) using a sterile alligator clip connector attached to the junction box, and were recorded on an EP Workmate™ version 4.3.2 system (Abbott Laboratories, Chicago, IL, USA). QLV was measured using techniques described by Singh et al.^9^ and Gold et al.^3^; namely, from the onset of the QRS complex to the peak of the first positive or negative deflection that was at least 50% of the total LV electrogram amplitude. Electrograms were recorded at multiple sites in those veins that were of clinical interest as potential targets for CRT pacing and were used to select the target vein for lead placement. After selection of a target pacing site, a permanent LV pacing lead was advanced over the wire and one of the electrodes was positioned at the same site from which the guidewire recording was obtained. Only those guidewire tracings that were recorded from the same site as the final LV lead electrode position were included in the analysis for this paper. Eighteen patients received quadripolar LV leads and two patients received bipolar LV leads. When QLV was measured from the implanted lead, the cathodal alligator clip was connected to the LV lead electrode that most closely reproduced the position of the guidewire under fluoroscopy, and the anodal alligator clip was connected to the surgical retractor. In seven patients, a separate pair of alligator clips was used to make simultaneous bipolar recordings from the RV lead.

This study was approved by the Institutional Review Board for Human Study Research at OhioHealth in Columbus, OH, USA and was performed in accordance with the ethical standards as laid down in the 1964 Declaration of Helsinki. Any requirement for patient consent was waived due to the retrospective nature of the study.

### Statistical analysis

The demographic and clinical characteristics of patients in this study were described using frequencies and percentages for categorical variables and using means and standard deviations (SDs) for continuous variables, respectively. QLV measurements and electrogram durations obtained with a guidewire and the permanent pacing lead at the same site prior to CRT implantation were plotted using scatter plots and evaluated using Pearson’s correlation and paired t-tests. Statistical significance was set at p < 0.05. A priori, assuming a type I error of 0.05, using a two-tailed paired t-test, we had greater than 95% power to detect a difference of 10 ms between the two measurements with our sample size of 20 patients, assuming a SD of 10 ms. The amount of time required to make QLV measurements using a guidewire, and the amount of fluoroscopy time used during CRT implantation, were reported using means and SDs, and medians and ranges, respectively.

## Results

In this study, a total of 20 subjects underwent implantation of a CRT-D (n = 12) or a CRT-P (n = 8) device. The demographic and clinical characteristics of these patients are shown in **Table 1**. The average ejection fraction was 36.5% ± 12.7%, and the average QRS duration was 164.0 ms ± 17.1 ms. The average amount of time required to make QLV measurements using the guidewire was 11.7 (SD: 6.8, median: 10.0, range: 2.0–26.0) minutes. The average amount of fluoroscopy time used during the CRT implantation was 10.9 (SD: 5.1, median: 9.5, range: 4 .0–22.0) minutes. This included insertion of right atrial, RV and LV leads for new implants. QLV measurements were made with the pacing lead only in the final position, and were compared with those that had been made with the guidewire at that site.

**Figure 1:**
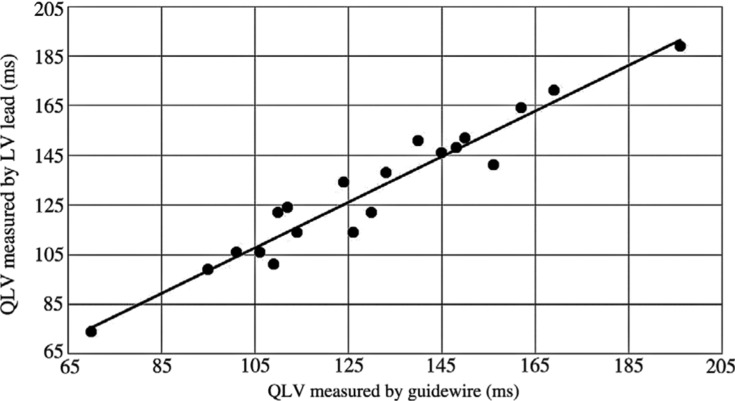
Scatterplot showing the relationship between QLV as measured by a guidewire and QLV as measured by the LV lead. QLV measurements obtained with a guidewire and the permanent LV pacing lead at the same site were strongly positively correlated according to results from Pearson’s correlation (r = 0.965; p < 0.001).

No difference in QLV measurements obtained with the guidewire (133.4 ms ± 27.1 ms) and the permanent pacing lead (133.8 ms ± 25.9 ms) placed at the same site was found (p = 0.569). QLV measurements obtained with a guidewire and the permanent LV pacing lead at the same site were strongly positively correlated (r = 0.965; p < 0.001) **(Figure 1)**. Overall, the mean absolute difference in QLV measurements was 6.0 ms (SD: 4.8 ms, median: 5.0 ms, range: 0.0–15.0 ms). There were no procedure-related complications and no occurrences of LV lead dislodgement. Eleven patients underwent a follow-up assessment of LV function at three months or more post-implantation. Nine of these 11 patients demonstrated an increase in LV ejection fraction of 10% or more at their respective follow-up times.

**Figure 2:**
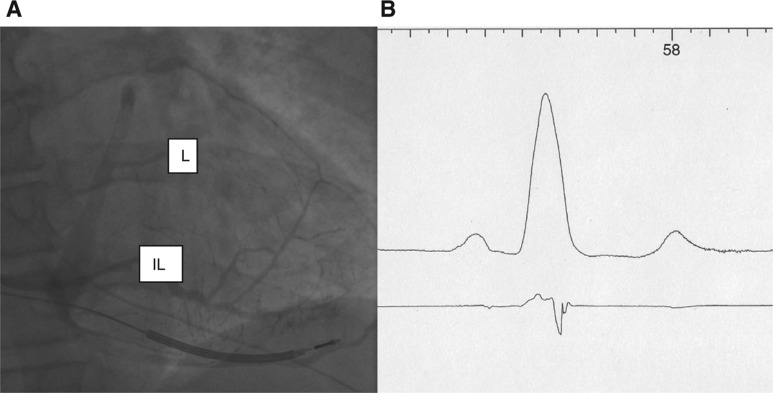
**A:** Retrograde venogram in right anterior oblique (RAO) projection after placement of the RV lead. A lateral vein (L) and an inferiorlateral vein (IL) are noted. **B:** Electrocardiogram (ECG) lead I and LV electrogram recorded when the guidewire was in the proximal portion of the L. The QLV measured at this site was 111 ms.

**Figure 3:**
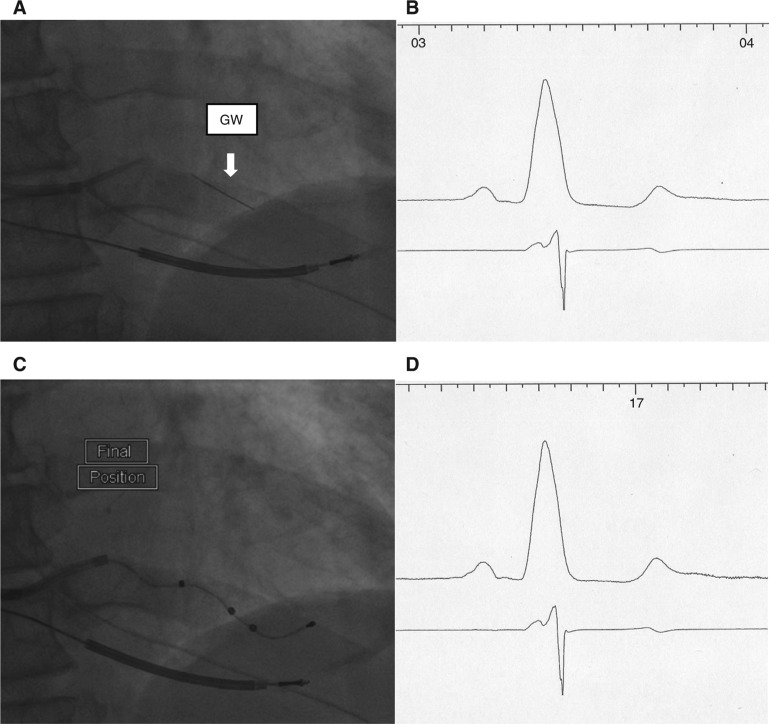
The same patient as shown in **Figure 2**. **A:** RAO fluoroscopy image showing the guidewire in the proximal portion of the IL. **B:** Surface ECG lead I and LV electrogram recorded from the guidewire in the position in **Figure 2A**. The QLV measured at this site was 138 ms. **C:** Quadripolar LV lead in the final position after being advanced over the guidewire.** D:** Surface ECG lead I and LV electrogram recorded from electrode P4 of the quadripolar lead. The QLV measured from this electrode was 124 ms.

**Figure 4:**
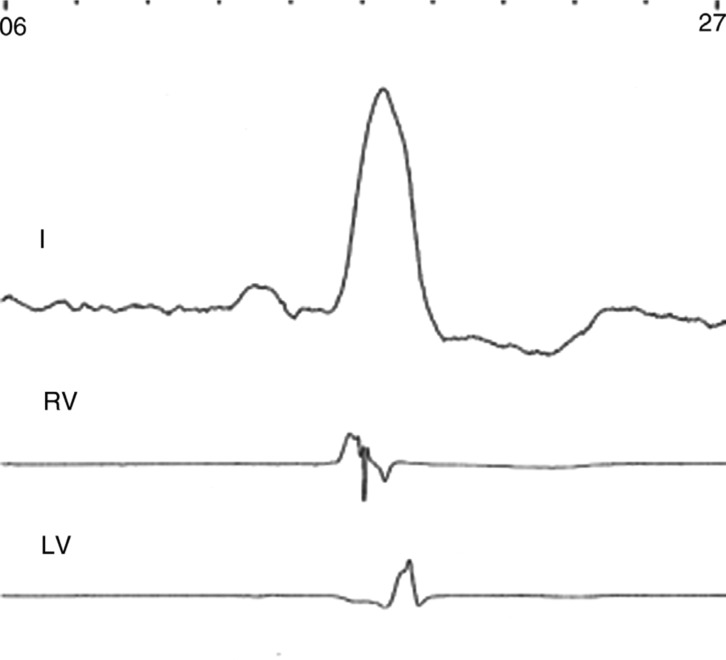
Surface ECG lead II, RV, and LV electrograms recorded at CRT-D implantation. The RV electrogram was recorded in a bipolar configuration from the RV lead. The LV electrogram was recorded from the guidewire as described in the text. QLV was 110 ms and the RV to LV interval was 66 ms.

**Figure 5:**
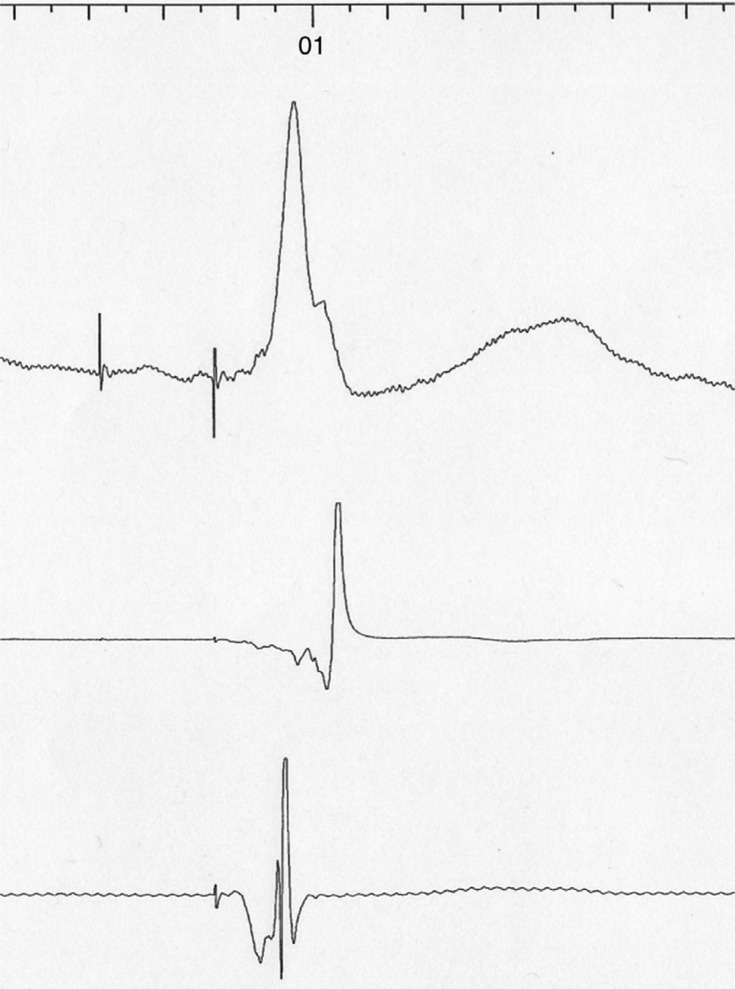
A surface ECG lead II, LV, and RV electrograms recorded from a pacemaker-dependent patient at the time of their upgrade to CRT-D shows atrioventricular pacing from the patient’s pre-existing permanent pacemaker. LV and RV electrograms recorded from newly implanted LV and RV leads are shown. The QLV measured from the LV lead was 162 ms. The RV to LV interval was 72 ms.

**Figure 6:**
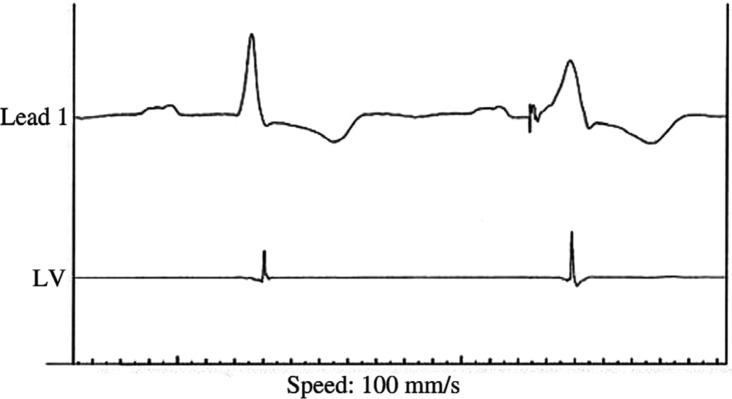
Surface ECG tracings and LV electrogram recorded from a patient with a prolonged PP interval and intermittent high grade atrioventricular block at the time of CRT implantation. The QLV of the conducted beat was 93 ms. The QLV of the RV paced beat was 132 ms.

Representative fluoroscopic images and electrogram tracings are shown in **Figures 2 through 6**. Retrograde venography in a patient undergoing implant of a CRT-D device demonstrated a high lateral and inferolateral vein **(Figure 2A)**. Positioning the guidewire in the proximal and distal aspects of each vein and then measuring the QLV at each site demonstrated the longest QLV (138 ms) to be in the proximal inferolateral vein **(Figures 2B and 3A to 3D)**. A quadripolar lead was then selected that was capable of concentrating electrodes at that site, and was positioned over the guidewire, achieving a QLV of 124 ms from electrode P4 **(Figure 3D)**. RV and LV electrograms were recorded in seven patients** (Figures 4 and 5)**, demonstrating the ability to measure RV to LV intervals in addition to QLV prior to insertion of the LV pacing lead. This approach was used primarily in patients with paced ventricular rhythm undergoing an upgrade from a permanent pacing device to a CRT-D **(Figure 5)**, and in patients with atrioventricular conduction diseased with preserved LV function undergoing the implantation of a CRT-P device **(Figure 6)**.

## Discussion

CRT is a key treatment option for patients with chronic heart failure, but the non-responder rate ranges from 20% to 40%.^2^ One of the strongest predictors of positive response to CRT is pacing from a site with prolonged QLV (> 95 ms).^3,4^ However, the site of longest QLV is variable, and is not always located in the proximal lateral wall.^10,11^ Identifying sites of prolonged QLV at implant allows for the selection of sites likely to be associated with a high response rate. Studies that have prospectively measured QLV have thus far used 3D mapping systems or have moved the permanent LV pacing lead from site to site.^5–8,12^ The present study describes a simplified method to measure QLV intervals using a unipolar guidewire during CRT implantation that does not require pairing with a 3D mapping system or repeated maneuvering of an LV pacing lead. The QLV measured with the guidewire corresponds closely with the value measured following placement of the lead. This provides a cost-effective alternative to the use of a 3D mapping system and a technically simpler approach to moving the LV lead from vein to vein.

Anatomic studies have shown that patients often have more than one potential target vessel for LV pacing.^10,13^ When more than one potential target vessel is available, coronary vein location, length, caliber, and tortuosity may influence the implanting physician’s assessment of which coronary vein is to be targeted and which LV lead will be selected. It is often easier to place a guidewire in a prospective target vein than to insert a permanent pacing lead. The guidewire method described in this study allows for implanting physicians to prospectively measure the QLV of target veins prior to investing the time and effort to position the permanent pacing lead, with confidence that it will closely correlate with the QLV measured from the pacing lead. It is likely that knowledge of the QLV, when added to other factors, will influence the selection of a target vein, the pacing site within that vein, and/or the lead to place in it.

Interventricular conduction delay can be assessed by measuring the time from RV to LV activation. Longer RV to LV interval duration has been shown to independently predict response to CRT, and exhibited a graded response that was present even in non-LBBB patients and in those with a QRS < 150 ms.^14^ This interval can be readily evaluated using an implanted RV lead and the guidewire technique as described in the present study. Measuring the RV–LV interval prior to anchoring either lead provides the implanting physician with the earliest opportunity to reposition either lead to a pacing site that is more likely to maximize the patient’s response to CRT. The guidewire method to measure local ventricular activation described here can also be used during RV paced rhythm, which may be beneficial since LV activation during RV paced rhythm is not necessarily the same as that during intrinsic LBBB.^15^ Small differences in measured QLV with the guidewire and the LV pacing lead may reflect the inability to exactly replicate the site at which the guidewire was located when the LV lead is positioned and/or the differences in electrode size between the guidewire and the LV lead. The current study used only guidewires from two manufacturers. Although not all guidewires allow for electrical recording or pacing from the tip, temporary pacing with interventional wires has been reported during percutaneous coronary intervention and transcatheter aortic valve replacement, respectively.^16,17^ The average time from insertion of the guidewire to insertion of the LV pacing lead was almost 12 minutes. This time included positioning of the guidewire, but was primarily comprised of the actions of labeling the fluoroscopic image, measuring the QLV on the recording system, and labeling the measurement for cross-reference with the fluoroscopic image.

A prior study has described the use of a unipolar guidewire to evaluate the ability to pace the LV from a prospective LV lead site, and to screen for phrenic nerve stimulation.^18^ The present study adds the observation that a guidewire can also be used to measure QLV prior to lead insertion and then be used for actual placement of the lead. An earlier study has also described the use of a guidewire to map QLV with a 3D mapping system.^7^ The present study demonstrates that a guidewire can be used to measure QLV without a 3D mapping system. This simplified approach should reduce procedural cost and would be applicable to implant centers that do not have access to 3D mapping systems.

### Study limitations

The present study observations are limited by having been performed by a single implanting physician at a single site. Additional multi-investigator research is needed to determine how readily this technique can be reproduced. Further, the data were not evaluated in a blinded manner, since the QLV measured from the guidewire was evaluated intraoperatively to help guide vein selection.

Notably, the purpose of the present study was to demonstrate the feasibility of measuring QLV at the time of CRT implantation using a guidewire and to compare such to measurements from an LV pacing lead positioned at the same site without the use of a 3D mapping system and without moving a pacing lead from vein to vein. This study was not designed or powered to follow patients for clinical response, since the relationship between QLV and CRT response rate is well established.^3,8^ The present study did not seek to identify the site of the longest QLV in each patient, but sites with a QLV > 95 ms were identified in 19 out of 20 patients. This study nonetheless demonstrates the feasibility of making QLV measurements without an electroanatomic mapping system and prior to the selection of the final LV pacing lead.

## Conclusions

In this study, a guidewire method for measuring QLV prior to LV lead selection and insertion is described that closely correlates with the QLV subsequently measured with an LV pacing lead. Target veins can be mapped quickly for sites of late electrical activation with acceptable fluoroscopy time and without the requirement of 3D mapping or the need to move a permanent LV pacing lead from site to site.

## Figures and Tables

**Table 1:** Demographic and Clinical Characteristics of Patients Who Underwent CRT Implantation

Demographic and Clinical Characteristics	Number of Patients (n = 20)
**Age in years, mean (SD)**	**68.2 (9.1) years**
**Sex, n (%)**	
Male	14 (70.0%)
Female	6 (30.0%)
**CRT type, n (%)**	
Defibrillator	12 (60.0%)
Pacemaker	8 (40.0%)
**Procedure type, n (%)**	
Initial	12 (60.0%)
Upgrade	8 (40.0%)
**Left ventricular ejection fraction in %, mean (SD)**	**36.5% (12.7%)**
**Etiologic cause of heart disease, n (%)**	
Ischemic	5 (25.0%)
Nonischemic	7 (35.0%)
Other	8 (40.0%)
**QRS morphology, n (%)**	
Paced	11 (55.0%)
Left ventricular bundle block	9 (45.0%)
**QRS duration in ms, mean (SD)**	**164.0 (17.1) ms**
**Number of veins tested, n (%)**	
One vein	11 (55.0%)
Two veins	8 (40.0%)
Three veins	1 (5.0%)
Ejection fraction three months post-implant in %, median (range)*	**55.0% (20.0% to 60.0%)**

*Data not available for 11 patients (n = 9).

## References

[1] Leyva F, Nisam S, Auricchio A (2014). 20 years of cardiac resynchronization therapy. J Am Coll Cardiol.

[2] Kandala J, Altman RK, Park MY, Singh JP (2012). Clinical, laboratory, and pacing predictors of CRT response. J Cardiovasc Transl Res.

[3] Gold MR, Birgersdotter-Green U, Singh JP (2011). The relationship between ventricular electrical delay and left ventricular remodeling with cardiac resynchronization therapy. Eur Heart J.

[4] Gold MR, Leman RB, Wold N, Sturdivant JL, Yu Y (2014). The effect of left ventricular electrical delay on the acute hemodynamic response with cardiac resynchronization therapy. J Cardiovasc Electrophysiol.

[5] Niazi I, Ryu K, Hood R, Choudhuri I, Akhtar M (2014). Threedimensional electroanatomic mapping of the coronary veins during cardiac resynchronization therapy implant: feasibility and possible applications. J Interven Card Electrophysiol.

[6] Rad MM, Blaauw Y, Dinh T (2014). Different regions of latest electrical activation during left bundle branch block and right ventricular pacing in cardiac resynchronization therapy patients determined by coronary venous electro-anatomic mapping. Eur J Heart Fail.

[7] Rad MM, Blaauw Y, Dinh T (2015). Left ventricular lead placement in the latest activated region guided by coronary venous electroanatomic mapping. Europace.

[8] Liang Y, Yu H, Zhou W (2015). Left ventricular lead placement targeted at the latest activated site guided by electrophysiological mapping in coronary sinus branches improves response to cardiac resynchronization therapy. J Cardiovasc Electrophysiol.

[9] Singh JP, Fan D, Heist EK (2006). Left ventricular lead electrical delay predicts response to cardiac resynchronization therapy. Heart Rhythm.

[10] Del Greco M, Maines M, Marini M (2017). Three-dimensional electroanatomic mapping system-enhanced cardiac resynchronization therapy device implantation: results from a multicenter registry. J Cardiovasc Electrophysiol.

[11] Spencer JH, Larson AA, Drake R, Iaizzo PA (2014). A detailed assessment of the human coronary venous system using contrast computed tomography of perfusion-fixed specimens. Heart Rhythm.

[12] Zanon F, Baracca E, Pastore G (2014). Determination of the longest intrapatient left ventricular electrical delay may predict acute hemodynamic improvement in patients after cardiac resynchronization therapy. Circ Arrhythm Electrophysiol.

[13] Polasek R, Skalsky I, Wichterle D (2014). High-density epicardial activation mapping to optimize the site for video-thoracoscopic left ventricular lead implant. J Cardiovasc Electrophysiol.

[14] Mafi Rad M, Blaauw Y, Dinh T (2014). Different regions of latest electrical activation during left bundle-branch block and right ventricular pacing in cardiac resynchronization therapy patients determined by coronary venous electro-anatomic mapping. Eur J Heart Fail.

[15] de Cock CC, Res JC, Hendriks ML, Allaart CP (2009). Usefulness of a pacing guidewire to facilitate left ventricular lead implantation in cardiac resynchronization therapy. Pacing Clin Electrophysiol.

[16] Mixon TA, Cross DS, Lawence ME, Gantt DS, Dehmer GJ (2004). Temporary coronary guidewire pacing during percutaneous coronary intervention. Catheter Cardiovasc Interv.

[17] Faurie B, Abdellaoui M, Wautot F (2016). Rapid pacing using the left ventricular guidewire: reviving an old technique to simplify BAV and TAVI procedures. Catheter Cardiovasc Interv.

[18] Gold MR, Singh JP, Ellenbogen KA (2016). Interventricular electrical delay is predictive of response to cardiac resynchronization therapy. JACC Clin Electrophysiol.

